# Supplementary material for: Predicting the connectivity of primate cortical networks from topological and spatial node properties
Source: BMC Syst Biol. 2007 Mar 8;1:16. doi: 10.1186/1752-0509-1-16 (PMC1831788; doi:10.1186/1752-0509-1-16)
Supplement: Additional file 1 — Analysis of C. elegans data. The file provides the results of a supplementary data analysis of the neuronal network of C. elegans, using the same network reconstruction approach as for primate cortical connectivity. [file 1752-0509-1-16-S1.doc]

**Additional file 1 – Analysis of *C. elegans* data**


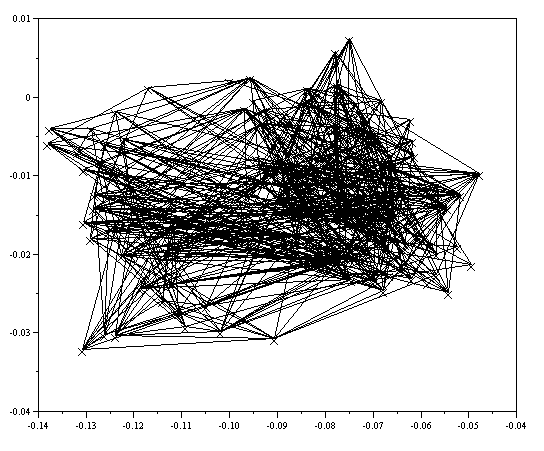


**Figure A1** – The neurons and interconnections in the *C. elegans* network. The figure was derived analogous to Figure 1.

Communities 1 and 2 include *N*_1_ = 67 and *N*_2_ = 64 nodes, respectively, and *E*_1_ = 604 and *E*_2_ = 534 directed edges. The clustering coefficients obtained for the two identified communities were found to be equal to 0.14 and 0.13, respectively. Table A1 gives the errors obtained while reconstructing these two communities by taking into account topological and geographical features. Figures A3 and A4 show the original and reconstructed matrices by considering topological (A3) and geographical (A4) features. It is evident from such results that rather poor reconstructions can be obtained for the *C. elegans* connectivity data.


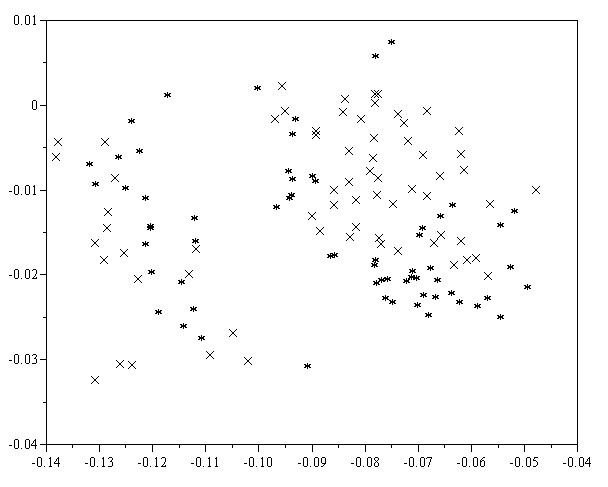


**Figure A2** – The two principal communities identified in the *C. elegans* network, represented analogous to Figure 3.

| **Network** | ***Hamm. Dist.*** | ***R1*** | ***R0*** | $\sqrt{R_{1}R_{0}}$ ***random*** | $\sqrt{R_{1}R_{0}}$ |
| --- | --- | --- | --- | --- | --- |
| **C. elegans / Comm. 1**  **Features reconstr.** | 1921 | 0.607 | 0.344 | 0.33 | 0.457 |
| **C. elegans / Comm. 1**  **Distance reconstr.** | 2007 | 0.301 | 0.592 | 0.33 | 0.422 |
| **C. elegans / Comm. 2**  **Feature reconstr.** | 1861 | 0.582 | 0.336 | 0.34 | 0.442 |
| **C. elegans / Comm. 2**  **Distance reconstr.** | 2225 | 0.311 | 0.482 | 0.34 | 0.387 |

Table A1 – Measurements comparing the original and reconstructed networks: ratio of overall matches, percentage of correct zeros, percentage of correct ones, and geometrical averages between the two latter percentages expected for random comparisons and obtained from the considered experiments.


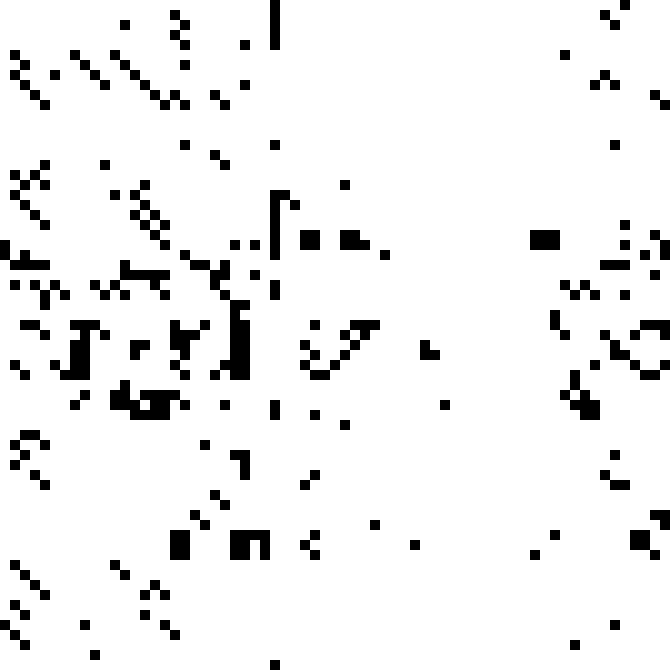

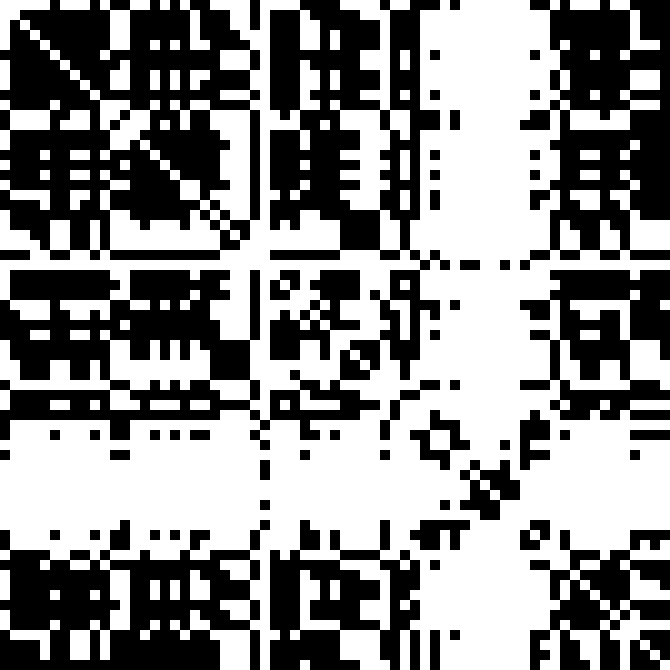


(a) (c)


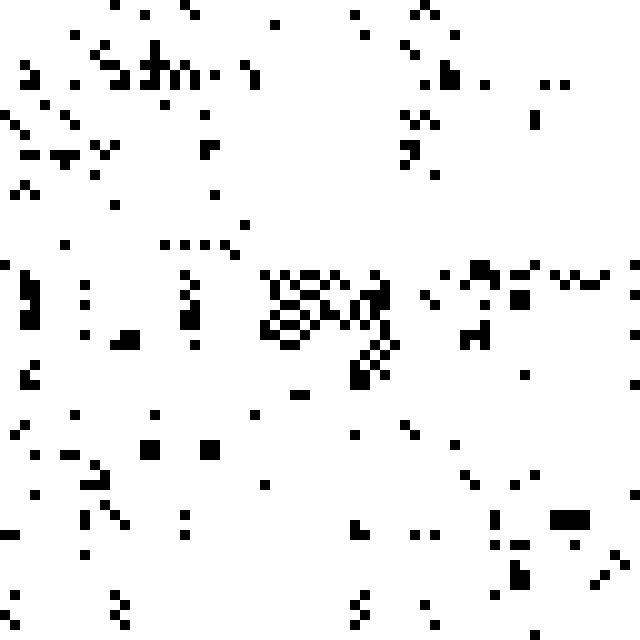

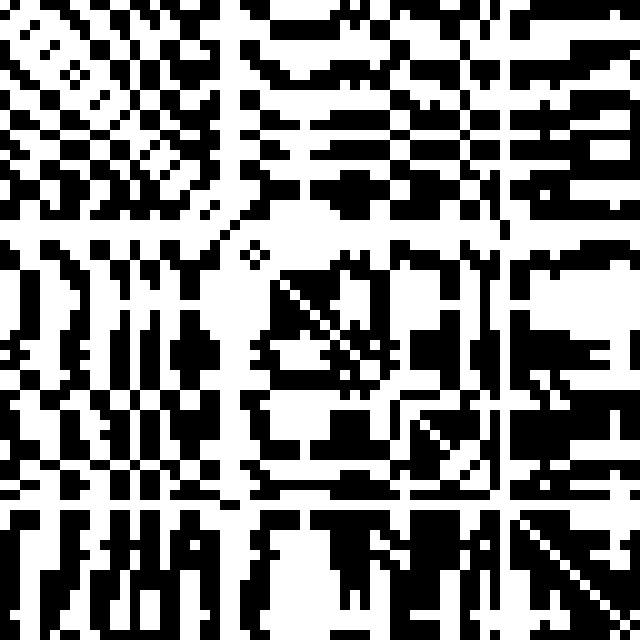


(b) (d)

**Figure A3** – The adjacency matrices obtained for the two *C. elegans* communities constructed with basis on the 4 topological features. The original two communities are shown in (a) and (b), while the respective reconstructions are presented in (c) and (d). Black points indicate presence of connections.


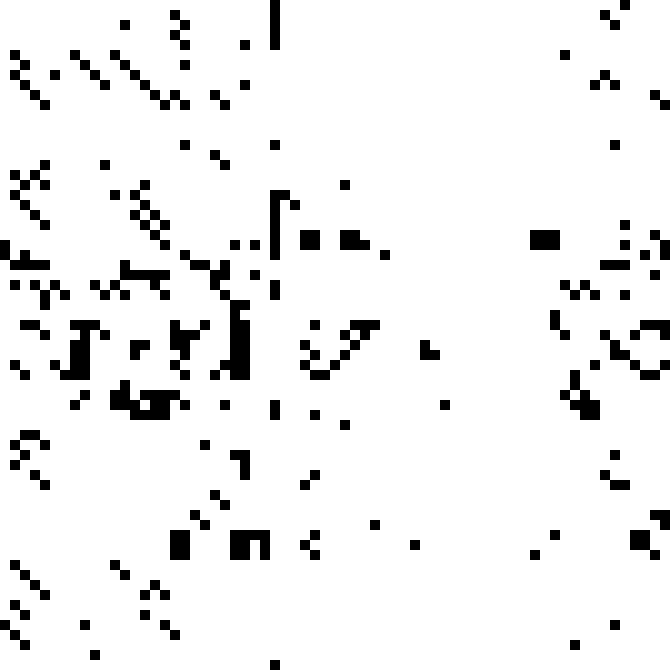

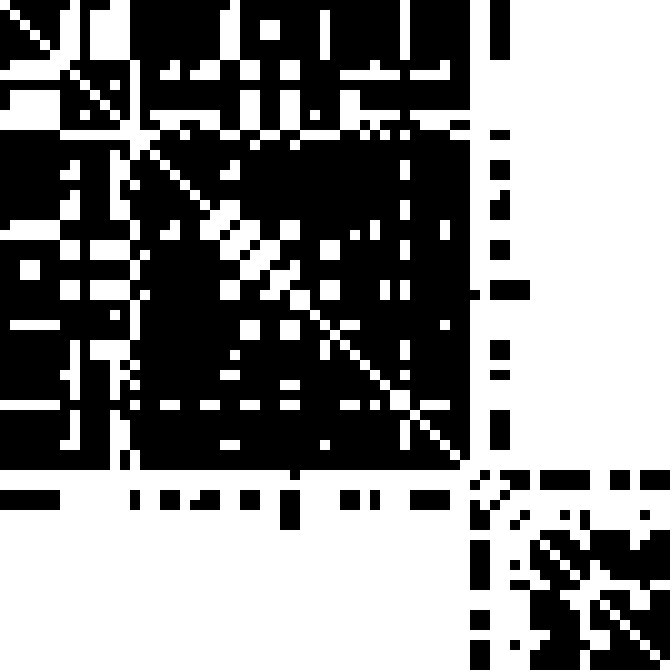


(a) (c)


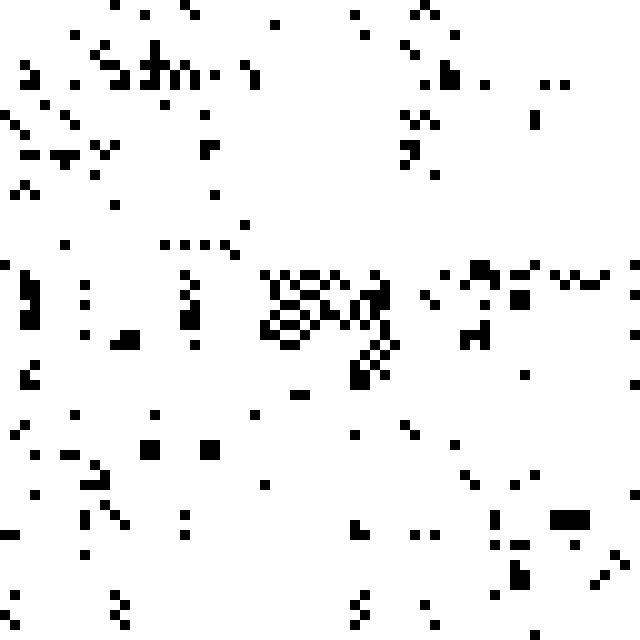

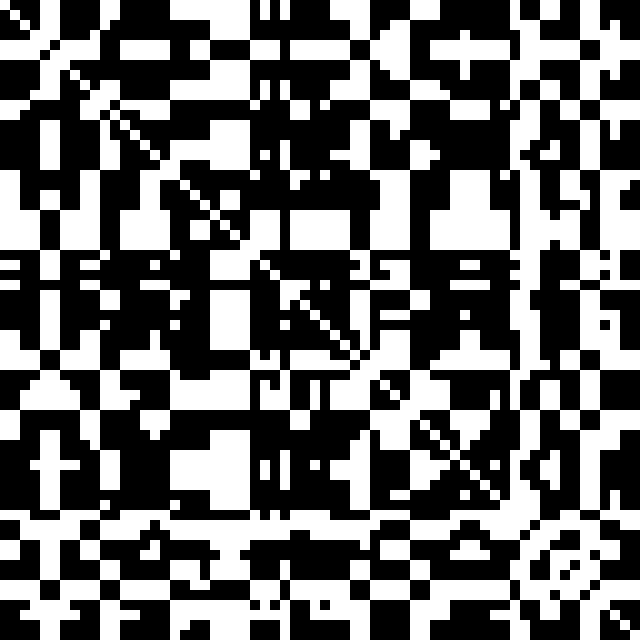


(b) (d)

**Figure A4** – The adjacency matrices obtained for the two *C. elegans* communities constructed with basis on the 3 geometrical features (recall that the area of each region is not applicable for this type of network). The original two communities are shown in (a) and (b), while the respective reconstructions are presented in (c) and (d). Black points indicate presence of connections.

# 
